# Three Cases of Rachitis

**Published:** 1873-01

**Authors:** Bernhard Grunhut, John C. Jay

**Affiliations:** Clinical Assistants; Clinical Assistants


### THREE CASES OF RACHITIS.

*Presented at Professor A. Jacobi's Clinic for Diseases of Women and Children, at College of Physicians and Surgeons, New York.*

[Reported by BERNHARD GRUNHUT, M.D., and JOHN C. JAY, Jr., M.D., Clinical Assistants.]

### CASE I.

*Clinical History.*—April 19, 1871.—Mary O'Neil, three years of age. Has been delicate from birth. For the last year, the child has been affected with diarrhœa, having four or five watery and slimy passages per day. Was eleven months old when it began to walk. Had whooping-cough about a year ago, and afterwards had scarlet fever. Child's first tooth appeared when it was eight months old.

*Remarks.*—The patient has a square, large rachitical head, with the anterior fontanelle yet open, which is very rare at this age. The liver is enlarged, and causes the child to complain of pain in that region. The ribs are soft and tender to the touch, rendering it necessary to be careful in handling her. The chest is characteristic of rachitis, there being flatness above and in front, together with flat sides, forming a quadrangular rather than an elliptical chest. The child has an osseous enlargement upon the fourth metacarpal bone of the left hand, and of the first phalanx of the little finger and thumb; also, upon the first phalanx of the great toe of the right foot. This is due to mal-development and softening of the bones; is mostly met with in young children and in their small bones, and comprises the disease commonly spoken of in the older text-books as cases of spina ventosa. The prognosis is good.

*Treatment.*—Patient was ordered to be given: Syr. phos. co., ʒ ss; ol. aselli jecoris, ʒ j. Three times a day. Also, to have a bath containing rock-salt twice a day, at first warm, and after a few days cold.

May 24, 1871.—Patient was again presented at the clinic. General condition much improved. The diarrhœa has ceased.

### CASE II.

*Clinical History.*—May 24, 1871.—John Blackwood, one year old. Was, when four or five months old, a fine-looking, fat baby. Child had night-sweats, especially about the head, so as to even wet the pillow. Its hair, which was very abundant at birth, is now very thin, the occipital portion of the head being almost bald. Is very costive. Its stools are hard, black, and often streaked with blood. Is said to pass from one to one and one-half teaspoonsful of fluid blood twice during the week. Child has to strain a great deal while at stool, and has a cough which began almost with its birth.

*Remarks.*—Professor Jacobi remarked that he had noticed the mother dwelling upon the fact that the child, when born, was remarkably fat and good-looking. This was very often the condition of rachitical children. Examining the fontanelle, it is found to be still open, and of the size that it should be when the child was seven months old, instead of being now nearly closed. The hemorrhages from which the child suffers are probably due to irritation produced by the hard fæces, but it should not be overlooked that they might be caused by polypi of the rectum, which are not very unfrequenty the cause of hemorrhage per anum in children. Upon digital examination of rectum, no polypus can be detected, but there are three or four prominences of the mucous membrane, which may become polypi by being twisted and turned with the fæces, and, as a consequence, assume the shape of pediculated polypi.

The child has been fed upon rice, which may in part account for the obstinate costiveness.

*Treatment.*—The diet is to be changed to farina and milk; the farina to be boiled for fifteen or twenty minutes with a little water and salt, and then mixed with equal parts of milk; also, to take beef-tea, mutton-broth, and syr. ferri. iod., gtt. x. Three times a day.

June 14, 1871.—Patient returned. Has one natural passage per day. General appearance greatly improved. Sleeps better, and is not so restless.

### CASE III.

*Clinical History.*—May 24, 1871.—Jesse Green, five months, brought to be cured of difficult respiration. Was very fat, but has fallen away. Cannot hold his head up. Has a swollen throat. Coughs; rolls his head during sleep; has no hair on the back of his head. Is costive. Has been reared on the "bottle" alone from birth, which fact is sufficient to account for rachitis.

*Remarks.*—Rachitis is a disease of assimilation and nutrition, and not of the osseous system. There is a deficiency in the development of the muscular tissue. There is hypertrophy of the liver and of the bronchial and tracheal glands, and the chronic bronchial catarrh, in this case, is probably due to the enlargement of the latter. This case has a very marked rachitical chest; the junction of the cartilages with the ribs is very prominent, and presents the ordinary rachitical diaphragmatic groove, which is the result of the yielding of the softened bones at the point of insertion of the diaphragm.

*Treatment.*—Is a general one. Fresh air; bathing in cold water (salt-water better); beef, eggs, milk, etc. Exercise to employ the muscles and to cause expansion of the lungs, and for these reasons the child should be kept out-of-doors as much as possible.

As regards medicines, we may choose from the following: Steel, iron, phosphates, and ol. morrhuæ.

This child was ordered to take: Syr. phos. co., ʒ ss. Three times a day.

June 21.—Mother reports child as improving.

[Remarks by the Editor.—The flattening of the sides of the chest, often marked, in rachitical children, is attributed to atmospheric pressure and the frequent lifting of the child beneath its arms. When this lateral flattening is excessive, we find the sternum pushed forward, giving rise to what is commonly known as "pigeon-breast;" the distance from the sternum to the spinal column in such cases is consequently increased. Sometimes there is a slight curvature of the spine (*kyphosis*), often evident at a very early period, and due to muscular laxity. The most marked feature of rachitis is thickening of the periosteum and enlargement of the epiphyses of the long bones, outward curvature of the tibia and fibula, and infractions of the long bones. Another peculiarity of rachitis is enlargement of the abdomen, caused by the relaxed state of the abdominal muscles, and the crowding down of the lungs of the diaphragm, and thus on the liver, spleen and intestines, the effect of which often causes congestion of the former, and, ultimately, fatty degeneration and enlargement of the liver, and enlargement only of the spleen.—B. F. D.]—*American Journal of Obstetrics.*

---

Use of the Chlorate of Potash to Prevent Abortion. Sir James Y. Simpson has used it in many instances, and recommends it in doses of ten to twenty grains three times a day, combined with the rest.

